# Supplementary material for: Inadvertent human genomic bycatch and intentional capture raise beneficial applications and ethical concerns with environmental DNA
Source: Nat Ecol Evol. 2023 May 15;7(6):873–88. doi: 10.1038/s41559-023-02056-2 (PMC10250199; doi:10.1038/s41559-023-02056-2)
Supplement: Supplementary file 1 — Supplementary Fig. 1. [file 41559_2023_2056_MOESM1_ESM.pdf]

nature ecology & evolution

Article https://doi.org/10.1038/s41559-023-02056-2

Inadvertent human genomic bycatch and intentional capture raise beneficial applications and ethical concerns with environmental DNA

In the format provided by the authors and unedited

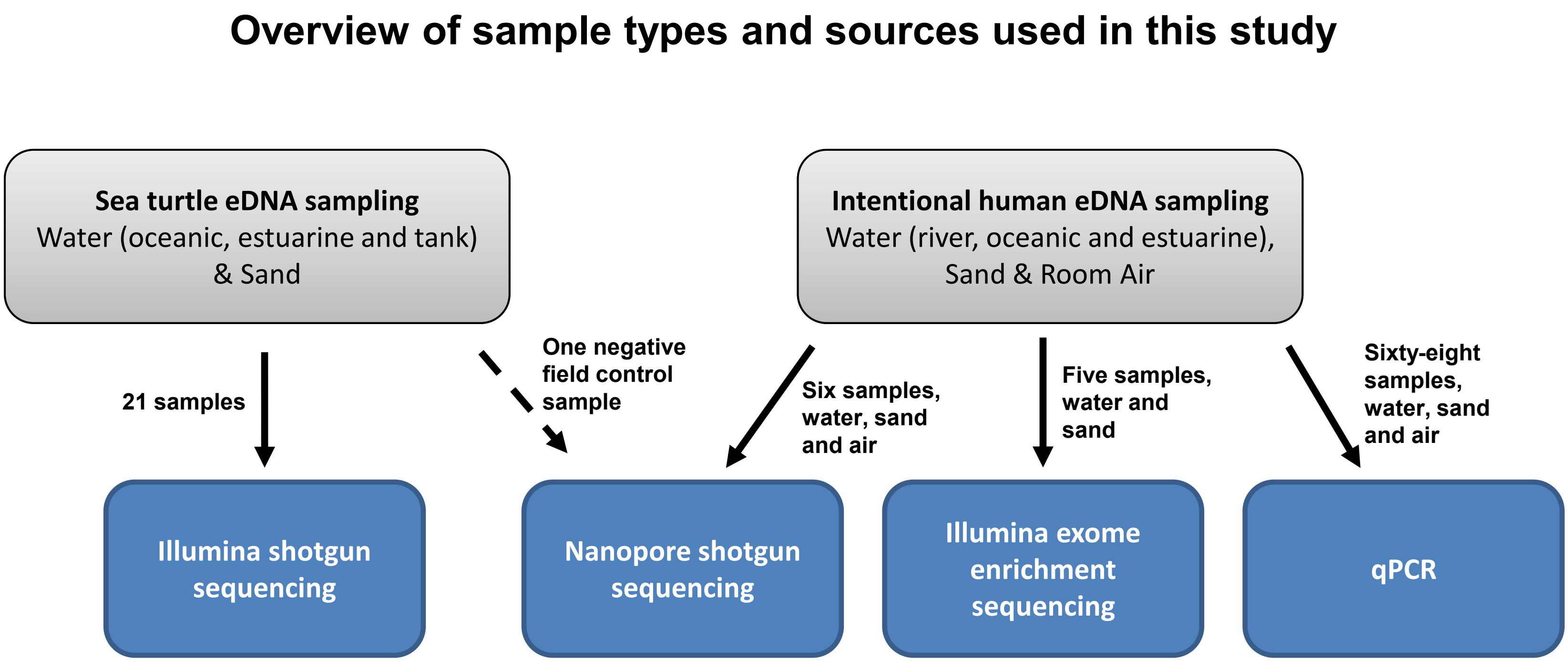

Supplemental Figure 1. Overview of the eDNA sample types and substrates and sources used in this study.
