# Supplementary material for: Inadvertent human genomic bycatch and intentional capture raise beneficial applications and ethical concerns with environmental DNA
Source: Nat Ecol Evol. 2023 May 15;7(6):873–88. doi: 10.1038/s41559-023-02056-2 (PMC10250199; doi:10.1038/s41559-023-02056-2)
Supplement: Supplementary file 2 — Reporting Summary [file 41559_2023_2056_MOESM2_ESM.pdf]

nature portfolio

Corresponding author(s): David Duffy

Last updated by author(s): Mar 13, 2023

Reporting Summary

Nature Portfolio wishes to improve the reproducibility of the work that we publish. This form provides structure for consistency and transparency in reporting. For further information on Nature Portfolio policies, see our Editorial Policies and the Editorial Policy Checklist.

Statistics

For all statistical analyses, confirm that the following items are present in the figure legend, table legend, main text, or Methods section.

n/a	Confirmed	
☐	☒	The exact sample size (*n*) for each experimental group/condition, given as a discrete number and unit of measurement
☐	☒	A statement on whether measurements were taken from distinct samples or whether the same sample was measured repeatedly
☒	☐	The statistical test(s) used AND whether they are one- or two-sided<br>*Only common tests should be described solely by name; describe more complex techniques in the Methods section.*
☒	☐	A description of all covariates tested
☒	☐	A description of any assumptions or corrections, such as tests of normality and adjustment for multiple comparisons
☒	☐	A full description of the statistical parameters including central tendency (e.g. means) or other basic estimates (e.g. regression coefficient) AND variation (e.g. standard deviation) or associated estimates of uncertainty (e.g. confidence intervals)
☒	☐	For null hypothesis testing, the test statistic (e.g. *F*, *t*, *r*) with confidence intervals, effect sizes, degrees of freedom and *P* value noted<br>*Give P values as exact values whenever suitable.*
☒	☐	For Bayesian analysis, information on the choice of priors and Markov chain Monte Carlo settings
☒	☐	For hierarchical and complex designs, identification of the appropriate level for tests and full reporting of outcomes
☒	☐	Estimates of effect sizes (e.g. Cohen's *d*, Pearson's *r*), indicating how they were calculated

Our web collection on statistics for biologists contains articles on many of the points above.

Software and code

Policy information about availability of computer code

Data collection	No software was used to collect research data. Supplemental Figure 1's number of shotgun eDNA publications was retrived from PubMed and Web of Science, as follows: Graph of “shotgun environmental DNA” papers in PubMed by year published. A literature search for “shotgun environmental DNA” returned 499 results in NCBI’s PubMed (https://pubmed.ncbi.nlm.nih.gov) and 654 results in Clarivate’s Web of Science (www.webofscience.com). Search conducted on March 11th 2022.
Data analysis	No custom software was used for analysis. All analysis was conducted using previously published software and tools, and referenced appropriately.

For manuscripts utilizing custom algorithms or software that are central to the research but not yet described in published literature, software must be made available to editors and reviewers. We strongly encourage code deposition in a community repository (e.g. GitHub). See the Nature Portfolio guidelines for submitting code & software for further information.

Data

Policy information about availability of data

All manuscripts must include a data availability statement. This statement should provide the following information, where applicable:

- Accession codes, unique identifiers, or web links for publicly available datasets
- A description of any restrictions on data availability
- For clinical datasets or third party data, please ensure that the statement adheres to our policy

All Illumina sequenced samples including raw reads are deposited in NCBI (https://www.ncbi.nlm.nih.gov/) under BioProject ID: PRJNA449022 (https://www.ncbi.nlm.nih.gov/bioproject/PRJNA449022). All Oxford Nanopore sequenced samples including raw reads are deposited in NCBI (https://www.ncbi.nlm.nih.gov/) under BioProject ID: PRJNA874696 (https://www.ncbi.nlm.nih.gov/bioproject/ PRJNA874696).

Field-specific reporting

Please select the one below that is the best fit for your research. If you are not sure, read the appropriate sections before making your selection.

- [ ] Life sciences
- [ ] Behavioural & social sciences
- [x] Ecological, evolutionary & environmental sciences

For a reference copy of the document with all sections, see nature.com/documents/nr-reporting-summary-flat.pdf

Ecological, evolutionary & environmental sciences study design

All studies must disclose on these points even when the disclosure is negative.

Study description	Observational study, no treatments. Assessed the number of human reads recovered from environmental DNA shotgun sequencing data. Additionally, collect water, sand and air samples eDNA from areas with high and low human habitation and quantified the level of human eDNA present with species-specific qPCR. Collected Oxford Nanopore long read sequencing of selected intentionally collected human eDNA samples (and Illumina Exome sequencing), including water from areas close to towns and human footprints in beach sand.
Research sample	Environmental DNA samples. Samples consisted of water eDNA (DNA recovered form sea water) or beach sand eDNA (DNA recovered form sea turtle nesting beaches).<br>Comprises of complex metagenomic data, not data purely from a single individual or species. Human originating DNA reads are reported here, sea turtle and pathogen DNA reads from these samples has been reported elsewhere (as referenced in the manuscript. Additionally, collected water eDNA samples form areas with high and low human activity, and human footprint beach sand samples and sand samples from an island with restricted human access. Air eDNA (air filter from rooms with or without humans present), human, sea turtle and turtle pathogen reads for air samples are reported here, as for air samples no turtle-related information was previously report, and as it demonstrates the feasibility of collecting data from multiple species and pathogens from the same air eDNA sample (which is potentially applicable to human medical settings also).
Sampling strategy	Illumina sequencing, sample sizes were dictated by the cost of high-depth shotgun sequencing (non-targeted). The maximum number of samples it was cost effective to sequence were analyzed. n = 20 NGS libraries. For qPCR analysis 68 eDNA samples were collected. Water eDNA was collected from a temperate and sub-tropical region to confirm the applicability of human eDNA in both climates, as there are known links between eDNA stability and climate type. Seven samples were used for Oxford Nanopore sequencing and five samples were used for Illumina exome sequencing, sample number primarily dictated by cost.
Data collection	All Illumina samples (shotgun and exome enriched) were sequenced at the University of Florida's (UF) Interdisciplinary Center for Biotechnology Research Core Facilities. All Nanopore samples were sequenced in the Duffy Lab at UF's Whitney Laboratory for Marine Bioscience. Water samples were collected by Jessica Farrell, David Duffy and Todd Osborne. Sand samples were collected by licensed nesting beach permit holders under the supervision of David Duffy or Jessica Farrell, Illumina samples). Human sand samples and air samples were collected by David Duffy in accordance with UF Institutional Review Board ethical approvals. Sand samples from Rattlesnake Island were collected by Jessica Farrell, David Duffy and Samantha Koda, with the permission and guidance of US NPS staff (Kurt Foote and Andrew Rich). qPCR was conducted by Jessica Farrell, David Duffy and Victoria Summers in the Duffy Lab at UF's Whitney Laboratory for Marine Bioscience, and by David Duffy in the Conway Core Facility, University College Dublin.
Timing and spatial scale	Illumina samples: Ad hoc. Initial set of 4 water samples (1 pooled tank library, and 4 wild water samples) were sequenced to confirm the viability of the approach (2017). Another set of 6 water samples, form more geographically diverse locations were sequenced (2021), and finally a set of sand samples (12 libraries) were sequenced. Sequencing was conducted in tandem with the optimization and development of sea turtle and pathogen qPCR assays. Water sampling sites range from northeast Florida to southern Florida (Florida Keys), all sequenced sand samples are from northeast Florida sea turtle nesting beaches, with the exception on one tank library and one rehab sand sample which were collected at the University of Florida's Whitney Laboratory for Marine Bioscience and Sea Turtle Hospital in northeast Florida.<br>qPCR & Nanopore samples: Intentional human eDNA sampling was conducted between May and July 2022 (water and sand) at sites in Northeast Florida, US and in East Ireland, room air eDNA sampling was conducted between Oct. and Nov. 2022 in Northeast Florida, US.
Data exclusions	No data were excluded from the analysis. Low quality reads were trimmed from each sample as part of the analysis pipeline, as is standard convention, but data form all sequenced samples are reported. Raw reads (prior to trimming) have also been publicly deposited, as is convention.
Reproducibility	Illumina & Nanopore samples: All samples represent individual replication, being independent of each other. No treatments etc. were conducted, these are observational data. Sequencing was done over 9 distinct library prep. and machine runs, on two different Illumina sequencers (HiSeq and NovaSeq) and a Nanopore MinION.<br>For qPCR, both biological and technical replicates were used. In addition, the human related eDNA water sampling was reproduced in both Florida and Ireland to confirm the reproducibility of this approach. Each sample set was analysed in its respective country, demonstrating reproducibility across labs and spatial scales.
Randomization	No groups were assigned. Samples were either water eDNA, sand eDNA or air eDNA depending on the environmental type originally samples. Illumina samples: Samples were random for human eDNA reads. Sequenced water samples were selected based on geographic spread and potential of the presence of sea turtles in a given location. Sand eDNA samples were selected based on sea turtle nesting activity, not human activity.<br>qPCR & Nanopore samples: Water sampling sites were selected based on their proximity to human habitation. Sand eDNA samples

	were either from human footprints or from non-footprint sand. Room air eDNA samples were either from rooms with or without humans present.
Blinding	Blinding was not performed, although the data was not collected solely for human eDNA applications (Illumina), and no human eDNA information was available to investigators prior to sequencing/qPCR and analysis (Illumina, qPCR and Nanopore).

Did the study involve field work? ☒ Yes ☐ No

Field work, collection and transport

Field conditions	Nesting beach sand samples were collected during the summer sea turtle nesting season in northeast Florida, during dry weather. Sand footprints and non-footprint sand samples were collected in the summer during dry weather.<br>Water samples were collected during in-water sea turtle surveying expeditions, and sampling trips to locations with high and low human habitation levels.
Location	State of Florida, location of sampling described in the manuscript and related sea turtle paper cited in the manuscript. For all (non-rehab) water samples, these were collect just below the surface (less than 1m depth). For all beach sand samples, these were collected by dragging the 50ml tube along the surface of the sand, or from nest spoil heaps.<br>The location of all intentional human eDNA sampling (Ireland and Florida) has been provided in the Supplemental Figures.
Access & import/export	No import/export permits were required as samples did not cross state or country lines. Samples were analyses in the country they were collected in. Permitting re the original samples is described in the sea turtle paper related to these samples, essentially: Sampling was carried out under permit number MTP-22-236 from the Florida Fish and Wildlife Conservation Commission (FWC) and with ethical approval for tissue sampling from the University of Florida's Institutional Animal Care and Use Committee (IACUC). Rehabilitation and nest patrol activities are conducted under FWC permit numbers MTP-21-228, MTP-21-103, MTP-21-084, MTP-21-029, MTP-21-041, MTP-21-140, MTP-21-023, MTP-21-046 and MTP-21-101 (rehabilitation and conservation activities were in no way impacted by this study). The Inwater Research Group conduct in-water sea turtle monitoring surveys under NMFS permit numbers 19528 and 16598 and FWC permit numbers MTP-18-125 and MTP-18-139. The Florida Hawksbill project conducts conduct in-water sea turtle monitoring surveys under NMFS permit number 22988 and FWC permit number MTP-21-077.<br>Human-related eDNA sampling was conducted with University of Florida Institutional Review Board (IRB-01) ethical approval under project number IRB202201336. Sampling at the Fort Matanzas National Monument (Rattlesnake Island) was conducted under a United States Department of the Interior National Park Service permit, permit number FOMA-2022-SCI-0003.
Disturbance	No disturbance was caused by the study as these are environmental samples. Water samples were obtained from rehab tanks while the turtle was not present. Sand samples were obtained after nesting events and after nest evaluations (conducted by already permitted nesting beach patrols). Human samples were obtained directly from the water (relatively small volumes) and from sand (relatively small volumes).

Reporting for specific materials, systems and methods

We require information from authors about some types of materials, experimental systems and methods used in many studies. Here, indicate whether each material, system or method listed is relevant to your study. If you are not sure if a list item applies to your research, read the appropriate section before selecting a response.

Materials & experimental systems

n/a	Involved in the study	
☒	☐	Antibodies
☒	☐	Eukaryotic cell lines
☒	☐	Palaeontology and archaeology
☒	☐	Animals and other organisms
☐	☒	Human research participants
☒	☐	Clinical data
☒	☐	Dual use research of concern

Methods

n/a	Involved in the study	
☒	☐	ChIP-seq
☒	☐	Flow cytometry
☒	☐	MRI-based neuroimaging

Human research participants

Policy information about studies involving human research participants

Population characteristics	Not applicable, as no population characteristics or personal information (beyond being male or female) was recorded from anonymous participants as it was not required due to the study design. The only criteria for inclusion was that the participants were human, and that they were capable of providing informed consent, and fully aware of qPCR and genomic sequencing technologies (as part of being able to provide fully informed consent). Human-related sampling was conducted with University of Florida Institutional Review Board (IRB-01) ethical approval under project number IRB202201336, with all participants providing informed consent. Four participants (three female and one male) provided sand footprint samples and six participants (five female and one male) provided room air samples (pooled room air).
Recruitment	No biases. Only the presence of and sequence of human eDNA was analysed, this was assessed from each footprint with no other correlating factors (i.e. no participant medical information or identifying information recorded). Voluntary participants

were sought at the institutional level. All participants providing informed consent. Participation was on a voluntary basis with no compensation received by the participants.

Ethics oversight	Human-related eDNA sampling was conducted with University of Florida Institutional Review Board (IRB-01) ethical approval under project number IRB202201336.

Note that full information on the approval of the study protocol must also be provided in the manuscript.
